# Scoping review of acute stroke care management and rehabilitation in low and middle-income countries

**DOI:** 10.1186/s12913-019-4654-4

**Published:** 2019-11-04

**Authors:** George Lameck Chimatiro, Anthea J. Rhoda

**Affiliations:** 1 University of the Western Cape, Cape Town, South Africa; 2 Medical Rehabilitation College, Box 256, Blantyre, Malawi

**Keywords:** Acute stroke, Stroke care structure, Stroke care process, Stroke care outcomes, Low and middle income-countries

## Abstract

**Background:**

Stroke is a major public health concern, affecting millions of people worldwide. Care of the condition however, remain inconsistent in developing countries. The purpose of this scoping review was to document evidence of stroke care and service delivery in low and middle-income countries to better inform development of a context-fit stroke model of care.

**Methods:**

An interpretative scoping literature review based on Arksey and O’Malley’s five-stage-process was executed. The following databases searched for literature published between 2010 and 2017; Cochrane Library, Credo Reference, Health Source: Nursing/Academic Edition, Science Direct, BioMed Central, Cumulative Index to Nursing and Allied Health Literature (CINNAHL), Academic Search Complete, and Google Scholar. Single combined search terms included acute stroke, stroke care, stroke rehabilitation, developing countries, low and middle-income countries.

**Results:**

A total of 177 references were identified. Twenty of them, published between 2010 and 2017, were included in the review. Applying the Donebedian Model of quality of care, seven dimensions of stroke-care structure, six dimensions of stroke care processes, and six dimensions of stroke care outcomes were identified. Structure of stroke care included availability of a stroke unit, an accident and emergency department, a multidisciplinary team, stroke specialists, neuroimaging, medication, and health care policies. Stroke care processes that emerged were assessment and diagnosis, referrals, intravenous thrombolysis, rehabilitation, and primary and secondary prevention strategies. Stroke-care outcomes included quality of stroke-care practice, functional independence level, length of stay, mortality, living at home, and institutionalization.

**Conclusions:**

There is lack of uniformity in the way stroke care is advanced in low and middle-income countries. This is reflected in the unsatisfactory stroke care structure, processes, and outcomes. There is a need for stroke care settings to adopt quality improvement strategies. Health ministry and governments need to decisively face stroke burden by setting policies that advance improved care of patients with stroke. Stroke Units and Recombinant Tissue Plasminogen Activator (rtPA) administration could be considered as both a structural and process necessity towards improvement of outcomes of patients with stroke in the LMICs.

## Background

Stroke is a major public health concern, both debilitating and deadly to millions of people worldwide each year [1]. Stroke frequently leads to loss of functional independence from physical and cognitive dysfunction. While stroke is stressful, efforts toward better outcomes, such as limiting the disability, more effective means of coping with loss, adaptation to change, and temporal ordering of coping processes could help patients recover to their highest possible functional level [2]. To achieve these outcomes, high quality care is needed. The quality of patient care can be conceptualized using a model, such as the one developed by Donebedian.

### Donabedian model (DM): the conceptual framework

Known as the most comprehensive model for assessing quality of health care [3], as presented in 1966, the model has three distinct aspects to health care quality: structures, processes, and outcomes (SPO) [4]. Accordingly, *Structures* of health care are defined as the physical and organizational aspects of care settings, for example, facilities, equipment, personnel, operational, and financial processes supporting medical care. *Processes* in patient care include resources, mechanisms provided by the health care structures to carry out patient-care activities that promote recovery, functional restoration, survival, and patient satisfaction (the *Outcomes*) [5].

In addition to developing the methodology for measuring structures, processes, and outcomes, Donabedian made an equally important contribution by prioritizing governance and management. When supported by measurement methods, they could be the determining causes of the effectiveness and efficiency of health services [6]. Related to quality of care, Donabedian said “systems … are enabling mechanisms only. It is the ethical dimension of individuals that is essential to a system’s success.” [7]. This is in sharp contrast to the ideologies of other researchers who advocated universal coverage or claimed that physicians’ clinical and financial autonomy was a precondition for the quality of care [6].

The relationships between structures, processes, and outcomes were effectively presented in a South African study which assessed quality of integrated chronic disease, among patients and operational managers. The study found that structure-related construct was the availability of equipment, supply of critical medicines, and accessibility of chronic disease care. Those related to process were communication between the nurses and patients, attendance of the nurses to patients’ health needs, professional conduct of the nurses, nurses’ friendliness with patients, hospital referrals for patients, pre-packaging of medicines, physical examination of patients; and the time nurses spent with patients during consultation. Whereas, outcome-related construct included coherence of integrated chronic disease care, competence of nurses, and patients’ confidence in nurses [8].

Effective stroke care included assessment (completed within 48 h post stroke) [9], early mobilization to prevent or reduce complications such as infections, deep vein thromboses (DVT), and falls, and promote recovery [10], avoidance of urinary catheterization, leading to bladder infections [11], treatment of hypoxia, hyperglycemia, and suspected infections [12], and ongoing rehabilitation activities [13]. Internationally, four acute-care intervention processes have been recommended by most stroke experts as the most effective front-line interventions. Those included care in a specialised stroke unit (SU), [14], thrombolytic therapy through tissue plasminogen activator (t-PA) for acute ischemic stroke (within 4.5 h of initial onset) [15, 16], aspirin therapy for acute ischaemic stroke (within 48 h) [17], and decompressive surgery (within 48 h) [18] to reduce stroke-related mortality and morbidity [19].

As highlighted, the most effective stroke-care service requires an organized setting such as SU care [14]. However, where units do not exist, patients with stroke should be admitted to generic wards, staffed by a coordinated multidisciplinary team with special expertise in stroke care [20]. In some settings in LMICs, such as those in Africa, multidisciplinary stroke-care teams are formed with non-specialist service providers, and in the absence of other equally important professionals, such as speech therapists [21]. There is also a need for readily available radiological equipment to make the most accurate stroke diagnosis [22]. Clinical methods predominate in resource-limited settings, [23], although Imam and Olorunfemi said such methods still need proper attention through a skills check [24]. Accurate diagnosis of stroke related to site, size, and type still requires radiological methods.

It was recommended that appropriate systems of stroke care be established in LMICs to control the increasing death and disability associated with stroke [25, 26]. However, implementation of those systems is challenged by the absence of direct health policy support from the state or national level for stroke care [27].

Applying Arskey and O’Malley’s 2005 scoping review methodology [28], the purpose of this review was to systematically document the evidence of stroke care and rehabilitation service provision in LMICs that could inform the development of a more-effective contextual model of care for Malawians and ultimately an improvement in their quality of life**.**

The following broad question was used to ensure that all relevant literature was included [28],

“What are the components of acute stroke care and rehabilitation services by health personnel across the hospital stroke care continuum in LMIC?” This question was delineated to have “stroke care” AND “profession and setting” search terms.

## Methods

### Identifying relevant studies

According to Joanna Briggs Institute [29], in searching for articles to be included in a scoping review, the researcher undertakes a limited search of relevant databases, followed by analysis of text words from titles, abstracts, and index words. Therefore, this researcher performed an electronic database search that included Cochrane Library, Credo Reference, Health Source: Nursing/Academic Edition, Science Direct, BioMed Central, CINNAHL with full text, Academic Search Complete, and Google Scholar in January 2018. Search tools such as medical subject headings (MESH) and truncation to narrow or expand searches were utilized. Single and combined search terms included acute stroke, stroke care, stroke rehabilitation, developing countries, and LMIC. This study included empirical English articles on stroke care, published between 2010 and 2017. Excluded studies were those from high-income countries using the World Bank definition [30] not within the defined range of publication time, and those covering participants under 18 years of age, per the United Nations Conventions of the Rights of the Child [31].

### Selection of articles for inclusion

The researchers used a two-stage selection criterion [32] (Halas, Schultz, Rothney, Goertzen, Wener, and Katz, (2014). Initially, the researcher and team (GC, SM, FM HZ, and JA) screened the titles and reviewed them for relevance to acute stroke hospital and rehabilitation care from LMICs. Secondly, they screened the chosen articles’ abstracts with key sections of the introduction for answers to the research question and objectives, methods that included the article design, setting or context, key findings, and conclusions.

### Data charting and collation

If the article met the inclusion criteria, the information relating to aim of the study was extracted and captured on a data charting form. The data charting table/form as displayed in Table 1 included the citation (author and year), country, recruitment context, study design, sample, outcomes based on objectives, and key findings. The table/form was peer-reviewed, with ambiguity removed in the process. The authors and research assistants (GC, HZ, JA, SM) initially extracted data independently, then met to determine if the data extractions were consistent with the aim of the study and the research questions (Table 1).

**Table 1:** Scoping review articles (key details)

Author reference & recruitment context	Study design	Sample	Content of interest	Key findings
Acute stroke presentation in hospitals				
1. Albertino, Joana, Ana, et,al. nd [33] Urban Hospitals. (Central hospitals, public hospitals, military hospitals & private clinics)	Retrospective & prospective August 1, 2005 - July 31, 2006	651 cases	Incidence, characteristics & short-term consequences of hospitalizations for stroke in Maputo and Mozambique	− 531 pts. (81.6%) with first stroke − 601 cases (92.3%) confirmed by CT scan (83.4%) or necropsy (8.9%) − 351 (58.4%) ischemic, 242 (40.3%) hemorrhagic, & 8 (1.3%) subarachnoid hemorrhage -Ischemic events increased continuously with age (higher in men 45 to 64 yrs) -Incidence of haemorrhagic stroke rose up to 74 years (yrs) of age; declining thereafter − 15% of stroke events occurred in subjects aged 45 yrs. (??and higher/lower/exactly) − 60% of pts. arrived at hospital on same day as symptom onset -Most prevalent risk factor was hypertension (86.6 to 96.0%) − 254 pts. (101 ischemic & 133 hemorrhagic stroke events) died during 28-day follow-up period
2. Yan, Li, Chen, et.al. 2016 [34]	Review 2015	17 included systematic reviews, observational cohort studies, meta-analyses, & case reports	-Risk factors for stroke -Primary prevention of stroke -Treatment of stroke during the acute stage -Secondary prevention -Stroke rehabilitation	- History of hypertension, current smoking, diabetes mellitis, diet risk score, physical inactivity, alcohol intake, psychosocial stress & depression, & cardiac causes -Guidelines for stroke diagnosis: patient history, physical examination, neurological examination & stroke scales, & diagnostic tests - Primary preventive approaches: blood pressure control & promotion & maintenance of healthy lifestyle: not starting to smoke, & smoking cessation for smokers, no binge drinking, being physically active, & a healthy diet with adequate fruit & vegetable intake, reduced dietary trans-fat intake, & reduced sodium intake -Intravenous thrombolysis approved as evidence-based treatment for acute ischemic stroke -Mechanical thrombectomy in combination with pharmacological thrombolysis improved functional outcomes - Pts. who received organized inpatient care in a SU were more likely to be alive, independent, & living at home 1 yr after stroke -Secondary prevention of stroke: blood pressure control, antiplatelet & lipid-lowering therapy, homocysteine-lowering therapy, self-management, & family support -Stroke rehabilitation: inpatient, home, & community-based programs, including physical, occupational, speech, & recreation therapies - Availability of & access to rehabilitation services low in LMICs -Factors for limited accessibility: poor physician knowledge of the role of rehabilitation; lack of rehabilitation components in the standard of care; long intervals from stroke onset to admission to rehabilitation; infrequent, unskilled, & short-lived provision of rehabilitation care; & inadequate public insurance or financial support for rehabilitation care
3. El Sayed, El Zahran & Tamim, 2014 [35] Urban hospital	Retrospective chart review	87 pts.	Pt characteristics & outcomes Barriers to rt-PA utilization	Mean age of 71.9 yrs.; most pts. arrived by private transport (85.1%): weakness & loss of speech most common presenting signs (56.3%); 37.9% of pts. presented within 4.5 h of symptom onset Nine pts. (10.3%) received rt-PA2 groups (rt-PA versus non rt-PA) with similar outcomes (mortality, symptomatic intracerebral hemorrhage, mRs scores, & residual deficit at hospital discharge) rt-PA utilization was higher than expected Delayed presentation barrier to rt-PA administration
4. Ashraf, Maneesh, Praveenkumar, et.al. 2015 [36] Urban Hospital. India	Cross-sectional prospective study (Jan- Dec 2012	264 pts.	Factors contributing to delay in hospital arrival	Median delay = 12 h. Distance from hospital, history of coronary artery disease, & presence of hemiplegia
5. Tirschwell, TGN, Ly, Van Ngo et.al. 2012 [37] Urban Hospital (*n* = 1) Vietnam	Prospective cohort study	754 pts.	Patient characteristics Clinical predictors of 28-day mortality for admitted pts.	328 (43.5%) ischemic, 356 (48.5%) hemorrhagic Risk factors: for ischemic stroke: atrial fibrillation, lower prevalence of hypertension, & previous history of stroke Pts. with ischemic stroke less likely to have disturbed consciousness & speech disturbances, likely to have observed weakness, lower mean systolic & diastolic blood pressures, & higher mean total cholesterol levels 28-day crude mortality was 20.3% for pts. with ischemic stroke & 51.0%???; overall 37% 28-day predictor of poor outcome: hemorrhagic stroke type, worse pre-stroke Modified Rankin Scale (mRS), disturbed consciousness, absence of observed weakness at presentation, higher diastolic blood pressure, higher glucose levels, current tobacco smoking, & history of hypercholesterolemia Strongest predictor: limited access to evidence-based standards of care due to limited local resources, & local evidence.
6. Robert & Zamzami, 2014 [38] Saudi Arabia	Literature review		Incidence, prevalence type, risk factors of stroke; influence of age, gender differences, neuropsychiatric manifestations, health-related quality of life (QOL), LOS (LOS), medical care, & rehabilitation.	Low incidence & prevalence of stroke compared to Western countries Ischaemic stroke predominated; Sub-Arachnoid Haemorrhage very rare Important risk factors: hypertension, diabetes mellitus, coronary diseases, & smoking Men at higher risk than women Depression not frequent Low QOL in pts. from Saudi Arabia compared with other countries Age & functional status influenced HRQOL Stroke severity, nature & other medical complications: predictors of LOS Ministry of Health offers rehabilitation services; have one active stroke center Research on stroke, establish SUs, increase public awareness, train health care providers, & increase the rehabilitation centers
7. Mohd Nordin, et.al. 2012 [39] Urban Hospital. (*n* = 1)	Retrospective study from June to October 2010 for pts. 2006–2009	557 pts.	Individuals receiving rehabilitation services, their functional status on discharge	1. Ischemic stroke highest stroke subtype (66.4%) 2. No research on causes of ischemic stroke in Malaysians 3. 62.7% received rehabilitation during hospitalization: daily physiotherapy & occupational therapy 4. 3.0 days mean time from diagnosis of stroke to initiation of rehabilitation 5. Mean disability level at discharge = 3.5 6. Highest level of function at discharge: walking, then standing from sitting, followed by bed mobility & sitting up from supine (47, 25, 15, 12%) 7. > 50% of pts. had mRS score > 3 on discharge 8. Inadequate rehabilitation services for acute & sub-acute stroke survivors
8. Badachi, Mathew, Prabhu et.al. nd [40] Tertiary care center, South India		100 consecutive acute ischaemic events	Failure of pts. to recognize stroke symptoms, awareness of thrombolysis as a treatment modality, failure of patient’s relative to recognize stroke,failure of primary care physician to recognize stroke,transport delay, lack of neuroimaging & thrombolysis facility in 1st hospital of arrival, & non-affordability	1. Poor recognition of stroke symptoms by pts., relatives, & primary health care physicians; hence prehospital delay; attributed to lack of knowledge of stroke symptoms & hesitation to initiate treatment 2. No facilities for neuroimaging & thrombolysis for most tertiary hospital 3. Low utilization of thrombolytic therapy due to high cost 4. Inadequate ambulance services, especially in rural areas 5. Education efforts & awareness 6. Training of emergency physicians 7. Improve infrastructure in district hospitals 8. r-TPA should be available at subsidized rates 9. Neuroimaging facilities should be improved & made affordable 10. Uses of dedicated ambulance services 11. Stroke prevention programs on thrombosis needed
Stroke care structure				
9. Ossou-Nguiet, Sossoumihen, Matali et.al. 2017 [41] Urban Hospital (*n* = 1)	Case report	1	Inadequate availability of IV thrombolysis & mechanical thrombectomy interventions in SU.	1. Pt received IV thrombolysis only & died 24-h after admission 2. The unavailability of mechanical thrombectomy interventions 3. Few radiologists & MRI in sub -Saharan Africa 4. Need for enough medical personnel & appropriate equipment in SUs
10. Linda, Sebastiana, & Vanessa, 2009 [42] Urban Hospital. (*n* = 1)	Retrospective study	195 pts. (94 admitted before initiating SU, 101 thereafter)	Outcome of multidisciplinary stroke care in limited-resource settings	1. Inpatient mortality dropped from 33% (*n* = 31) to 16% (*n* = 16) 2. Referral to inpatient rehabilitation increased from 5% (*n* = 53) to 19% (*n* = 513) at discharge 3. Standardized investigation and evaluation of stroke admissions was better 4. Initiation of secondary prevention strategies was achieved 5. Prevention & treatment of complications of stroke was also achieved 6. Evaluation by multidisciplinary rehabilitation team was better achieved in SUs than in general ward 7. Staff education in stroke care & standardized discharge & rehabilitation planning was also better achieved in SU, compared to general wards 8. Involvement of relatives in SU was better achieved than in general wards
11. Gould, Asare, Akpalu et.al. nd [43] Ghana			Review of local services for stroke care, assist with service plans, provide multidisciplinary education & training, & practical case-based problem solving	1. Need for frontline staff to improve delivery of stroke care 2. Establishment of Ghana’s first SU
12. Donia et.al. 2017 [44] Urban Hospital. Egypt	Case report (Oct, 2016)	1	Administration of rTPA, dose, recommended administration time & availability of 2 in hospitals	Partial improvement during 3-month period after rTPA administration
Stroke Care Process				
13. Leonard, Michael, George et.al. 2017 [45] Urban hospitals. (*n* = 11) A non-probabilistic purposive sampling technique used for recruitment	descriptive study: Nov 2015 - Apr 2016	11 participants (neurologists & physician specialists) & medical officers (general physicians).	evaluating available acute stroke services	1. Availability of designated accident & emergency departments 2. Limited functional diagnostic & assessment services 3. Specific stroke clinical guidelines in all study hospitals 4. Few SUs available 5. No functional & standardised multidisciplinary team was evident 6. No provision of thrombolysis using tPA reported 7. Surgical procedures not conducted in any study hospital 8. Fewer/absence of stroke specialist 9. No direct health policy support from the state or national level for stroke care
14. Clarke, 2013 [46]	Review of stroke evidence	I reviewer	Effect of multidisciplinary stroke care & possible future direction	1. Pre-hospital & acute services improved recognition of stroke, established rapid specialist assessment resulting in more accurate diagnose & quality care 2. Thrombolysis can be safely administered up to six hours after witnessed stroke onset 3. Rehabilitation is key to stroke care 4. Rehabilitation interventions remain limited in some areas 5. Rehabilitation should commence as early as possible after stroke 6. SU team includes physiotherapists, occupational therapists, speech & language therapists, stroke physicians, nurses & healthcare assistants 7. Multidisciplinary team care resulted in long-term reductions in death, dependency & need for institutional care, facilitate earlier discharge to home increases likelihood pts. will regain independence in activities supporting daily living, & result in fewer pts. requiring long-term institutional care 8. Multidisciplinary team approach most effective way of providing high-quality stroke services 9. Need for early referral to clinical psychologists or psychiatrists to provide interventions when need arises 10. Co-ordinated multidisciplinary teamwork made improvements in quality of care in pts. with stroke
15. Al Khathaami, Algahtani, Alwabel, Alosherey, Kojan, & Aljumah et.al. 2011 [47] Urban hospital, Saudi Arabia	Situation analysis study. Phone interviews.	83 Neurologist.	Views & beliefs about current stroke care in the country, system deficiencies, attitude towards t-PA use, logistics required to provide optimal stroke care, & the priorities needed for improvement	1. 71% of neurologists rated the care provided to stroke pts. at 6 or below on a scale between 1 (very poor) & 10 (best care) 2. Deficiencies in stroke care starting from prevention & education at the community level to post-stroke rehabilitation 3. Lack of thrombolysis program to the existing shortage of resources 4. Need for improvement
16. Ogungbo, Ushewokunze, Mendelow et.al. 2005 [48]. Nigeria	Situation analysis	Not specified	How to improve management of stroke	1. Population strategy by implementing Public awareness programs, Life style modification strategies 2. Introduction of stroke study groups & development of local guidelines among physicians
17. Rahil, Afshin, Anahid et.al. 2012 [49]	Letter to the Editor. Iran		Cost-effectiveness of rTPA in developing countries	1. No studies on cost-effectiveness of rTPA in developing countries 2. Studies done revealed rTPA as cost-effective in developed countries 3. Few rehabilitation centres in developing countries
Stroke care outcomes				
18. Baatiema, Chan, Sav et.al. 2017 [50]. Hospitals across Africa	Systematic review using PRISMA	4 non experimental studies with 330 study participants between 2009 & 2016	Clinical efficacy of SUs Thrombolytic therapy	2. Less deaths (16%) Vs 33% prior SU 3. LOS 6.8 days compared to 5.1 days i general wards 4. Stroke referrals to inpatient rehab was higher than to general ward, (19% vs 5%) 5. Though not significant, patient access to CT brain scan was higher, 16%, in SU compared to 13% in the general medical ward 6. Improved pt. outcomes with use of thrombolytic therapy 7. Thrombolytic therapy can generate optimal patient outcomes in Africa 8. Imperative for policymakers to increase efforts to increase the use of thrombolytic therapy in hospital settings to reduce the current disproportionately high stroke burden in Africa
19. Rhoda A, Cunningham N, Azaria, 2015 [51] Hospitals (*n* = 3) R, South Africa & TZ	Retrospective	452 pts.	-Time from stroke onset to admission -in-patient physio & rehabilitation	Time interval stroke onset to admission 6.8, 0.3, & 1.2 days LOS stay; 8.2, 7.38, & 12.19 for R, SA, & TZ respectively 40,68%, & 98% of pts. with stroke in R, TZ, & SA respectively, received physio-rehabilitation, 2 sessions/week in R & TZ, & 3 in SA
20. Olaleye & Lawal, 2017 [52] (Urban hospital Nigeria)	Retrospective	783 pts.	Inpatient physio rehabilitation	1. Mean LOS = 16.2 days 2. Referral rate for PT high (75.8%) 3. Mean time from admission to referral for PT = 3 days 4. Majority (63.4%) of pts. referred utilized PT; mean number of PT sessions during in-patient care = 8.7 5. Utilization of in-patient PT significantly associated with reduced LOS

### Collating, summarizing and reporting findings

To evaluate the evidence of stroke care in LMICs, the researchers summarized and categorized the data in line with the SPO model as the conceptual framework.

## Results

**Fig. 1:**
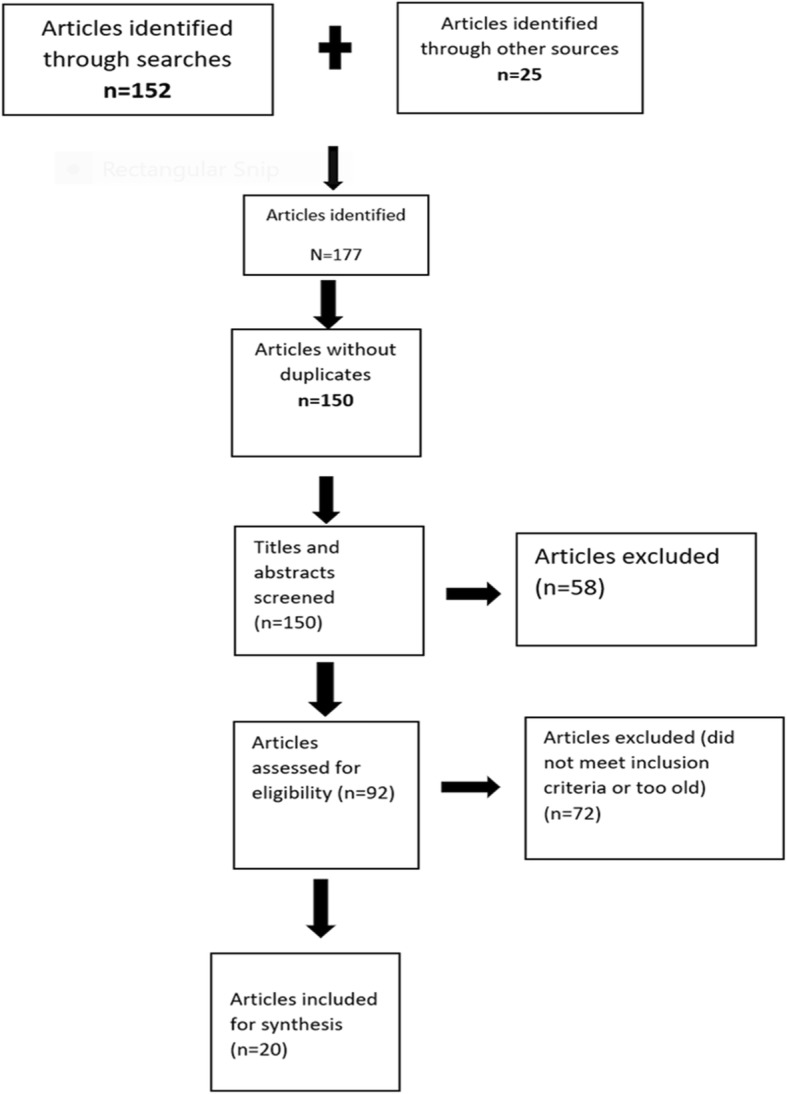
Scoping review process

The search identified 177 articles. Duplicates (*n* = 27) were manually removed. Title and abstract searches revealed 58 articles not within the context of the research question. Seventy-two articles did not meet the inclusion criteria for various reasons, leaving 20 articles for the scoping review (Fig. 1).

Evaluating the evidence on stroke care in the LMICs was facilitated by synthesizing findings of studies describing hospital acute stroke care and rehabilitation. The findings have been displayed, with their general characteristics such as the settings and locations. (Table 1). Various stroke care practices and service limitations have been noted based on conclusions and recommendation towards better practice by the authors. The Donabedian Model identified three primary themes of stroke care which are stroke care structure, process, and outcomes.

### Descriptive presentation of results

The overall aim of this study was to determine the current evidence of stroke care and rehabilitation in LMICs. To achieve this aim, we first needed to understand current stroke care services in the countries that share similar evidence of stroke burden and economic characteristics. Studies from LMICs per the World Bank classification from 2009 and 2017 were included. In addition to noting the number of articles, we recorded general characteristics such as study settings. The findings were further illustrated in three key thematic areas based on the Donabedian model. The results are highlighted by the combination of texts, tables, and figures.

### General characteristics of included articles

There was a total of 20 articles, published between 2009 and 2017. They were from different LMICc, with half [10] of the articles from Africa and the other half [10] from Asia. The design for most of the articles was a retrospective chart review (*n* = 8). Others were systematic reviews (*n* = 3), case report, situational analysis, and descriptive studies (*n* = 2), and 1 each for cross-sectional prospective study, literature review, descriptive studies, prospective follow-up. The focci of the articles was hospital care and rehabilitation services. The topics were categorized into SPO.

### Stroke care structures

This scoping review had 19 dimensions of stroke care, thematically established from 11 articles [33, 34, 38, 40–42, 45–48, 50]. Seven of them constituted the stroke care structure (Fig. 1). They were SU, accident and emergency department, multidisciplinary team, stroke specialists, neuroimaging, medication, and health care policies. There were few or no SUs in any of the settings within the LMICs [47]. The availability of designated accident and emergency departments that triage patients in LMICs, with specific clinical guidelines and functional and standardized multidisciplinary teams was associated with optimal stroke-care practice [45]. There are, however, few specialist health care workforces for acute stroke care both in number and variety [45] to constitute multidisciplinary teams. This type of teamwork was reported as the most effective way of providing high-quality stroke services, even in less-resourced LMICs [46]. There were no facilities for neuroimaging and thrombolysis for many tertiary hospitals, inadequate ambulance services especially in rural areas, limited educational efforts and awareness, inadequate training of emergency care physicians [40], unavailability of mechanical thrombectomy interventions, and few radiologists and MRI technology in sub -Saharan Africa [41]. In those countries that had key radiological devices, such Computed Tomography (CT) scanners, there were effective diagnostic workups on patients with stroke. For example, 601 cases (92.3%) were confirmed either by CT scan (83.4%) or necropsy (8.9%) as ischemic or hemorrhagic stroke, or subarachnoid hemorrhages [33]. Of note was that standardized investigation and evaluation of stroke assessment and admissions was much better after initiating multidisciplinary Stroke-Unit care, as established in some countries such as Ghana as a new development in the stroke care structure [45]. There was also limitation in availability of key medical supplies, such as rtPA in most LMICs [50]. There was poor physician knowledge of the role of rehabilitation; lack of rehabilitation components in the standard of care; a long interval from stroke onset to admission to rehabilitation; infrequent, unskilled, and short-lived provision of rehabilitation care; and inadequate public insurance or financial support for rehabilitation care [34].

In a related matter, countries such as Ghana had no direct health policy support from the state or national level for stroke care [45]. Studies recommended that policymakers could facilitate the utilization of thrombolytic therapy in hospital settings to reduce the current disproportionately high stroke burden in Africa [33]; rtPA could, therefore, be made available at subsidized rates; neuroimaging facilities could be improved and made affordable, in addition to use of dedicated ambulance services [40], and stroke prevention programs on thrombosis should be initiated [48]. Population strategy could focus on implementing public awareness programs, life-style modification strategies, the introduction of stroke study groups, development of local guidelines among physicians [48], upscaling rehabilitation services by training health care providers, and increasing the number of rehabilitation centers [38].

Some studies recommended a need for ministries of health and governments in LMICs to adopt and implement SUs for their reported impact on stroke outcomes [41, 42, 50]. For instance, the multidisciplinary teams afforded by SUs had been reported to have led to a drop in inpatient mortality, and increased referral to inpatient rehabilitation [42]. Additionally, standardized investigation and evaluation of stroke admissions was much better after initiating multidisciplinary SU care; initiation of secondary prevention strategies, prevention and treatment of complications of stroke, evaluation by a multidisciplinary rehabilitation team, staff education in stroke care and standardized discharge and rehabilitation planning and involvement of relatives were more easily achieved in SUs than in general wards [42, 50]. Introduction of SUs was also associated with reduced LOS [50].

### Stroke care process

**Fig. 2:**
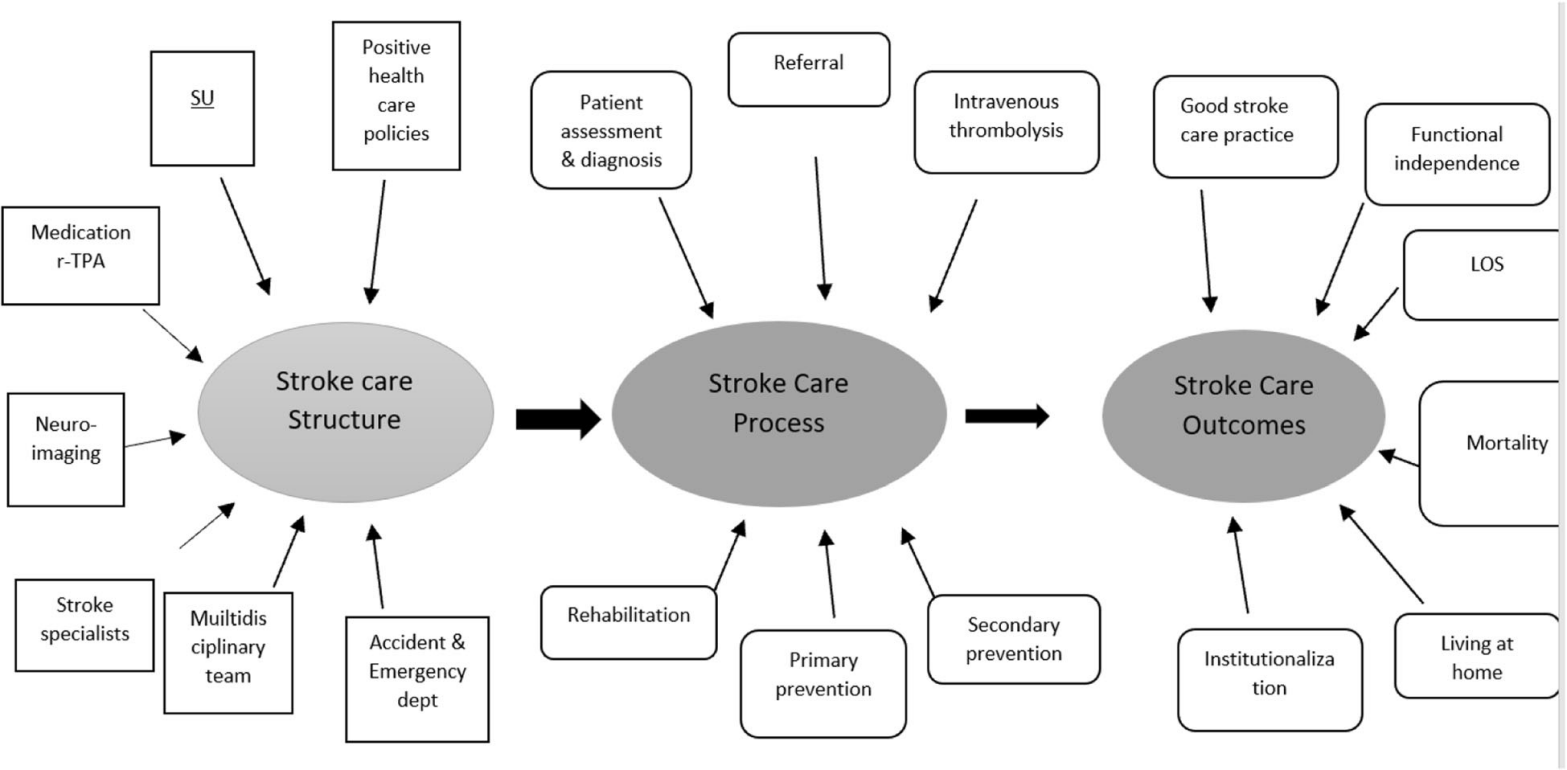
dimensions of care

The process of stroke was illustrated by 12 articles [33–36, 40–42, 46, 47, 49–52]. Six stroke care processes, illustrated in Fig. 2 consist of assessment and diagnosis, referral, intravenous thrombolysis, rehabilitation, and primary and secondary prevention strategies. Stroke assessment and diagnosis in LMICs was challenged by lack of proper stroke care structure [40, 41]. Stroke assessment for diagnosis included patient history, physical examination, neurological examination and stroke scales, and diagnostic tests [34]. However, recognition of stroke had improved following the introduction of pre-hospital and acute services [36].

Five studies highlighted the management of ischemic stroke using rtPA. Intravenous thrombolysis was approved as an evidence-based treatment for acute ischemic stroke in some settings within the LMICs [34]. Although, there were no studies on the cost-effectiveness of rtPA in developing countries, studies in developed countries revealed that rtPA was cost-effective [49] in treating ischemic stroke. Improved patient outcomes with the use of thrombolytic therapy could generate optimal patient outcomes in Africa [50], as lack of thrombolysis program was said to add to the existing shortage of resources [47]. Thrombolysis could be safely administered up to 6 hours after witnessed stroke onset [46]. However, delayed presentation to the hospital was the main barrier to rt-PA administration in Lebanon [46].

Rehabilitation services for patients with stroke was highlighted in seven articles. Of importance was the common finding that rehabilitation represented a key part of stroke care for the majority of patients. However, despite much research revealing the necessity of rehabilitation, the intervention remained limited in most LMICs. Recommendations were for rehabilitation to commence as early as possible after a witnessed stroke. SU team or just stroke team (in absence of SU) included physiotherapists, occupational therapists, speech and language therapists, stroke physicians, nurses and healthcare assistants [46]. Referral to in-patient rehabilitation varied depending on the approaches to stroke care, as introduction of SUs and multidisciplinary team in some LMICs was associated with a huge increase in referrals. The mean time from admission to referral for physiotherapy was 3 days [42]. The majority of patients referred utilized physiotherapy and the mean number of physiotherapy sessions received during in-patient care in the sampled urban hospitals varied. For example, physiotherapy was utilized 2 sessions per week in Nigeria [52], 2 sessions per week in Rwanda and Tanzania respectively, and 3 in South Africa [51].

Two reports highlighted stroke prevention strategies, with approaches including blood pressure control, promotion and maintenance of healthy lifestyles (i.e., not smoking or smoking cessation for smokers, no binge drinking, being physically active, and a healthy diet with adequate fruit and vegetable intake, reduced dietary trans-fats, and reduced sodium [33]. Secondary prevention of stroke, on the other hand, included blood pressure control, antiplatelet and lipid-lowering therapy, homocysteine-lowering therapy, and self-management and family support [34].

### Stroke care outcomes

Regarding stroke care outcomes, 7 studies (34, 35, 39, 41, 47, 49) reported a total of six dimension of stroke-care outcomes, illustrated on the right side of Fig. 1. They were quality of stroke care practice, functional independence, length of hospital stay (LOS), mortality, living at home, and institutionalization. Of note, was LMICs with better structure and processes of care such as availability of SU, patients were more likely to be alive, independent, and living at home 1 year after stroke [34]. On the other end, LMICs with no requisites had unsatisfactory or poor stroke service delivery [47] and poor stroke care practice [45]. Thrombolysis [45], mechanical thrombectomy in combination with pharmacological thrombolysis when indicated improved functional outcomes [34]. Utilization of in-patient physiotherapy (PT) was significantly associated with reduced LOS [52], and reduced mortality [38]. Absence of rehabilitation on the other hand was associated with reduced level of function, evidenced by increased modified Rankin scale (mRs) score [39]. Studies had further shown that a structural dimension such as multidisciplinary stroke care teams resulted in long-term reductions in death, dependency and the need for institutional care, facilitated earlier discharge to the home, increased the likelihood that patients would regain independence in activities that support daily living, and resulted in fewer patients requiring long-term institutional care in some settings within LMIC [46].

## Discussion

In this paper, we extracted data from twenty articles that highlighted stroke care in LMICs with the guide of the DM on quality of care as a conceptual framework. Nineteen dimensions of stroke care emerged, covering stroke-care SPO. Seven of the 19 dimensions that emerged were structural. They included a SU, accident and emergency department, multidisciplinary team, stroke specialists, neuroimaging, medication, and health care policies [33, 34, 38, 40–42, 45–48, 50]. Structurally, this review showed that there were few or no SUs in various settings within LMICs. As a result, stroke service delivery in those areas was unsatisfactory or poor [47]. The development and running of SUs was a key recommendation in most countries including the developing ones [21]. In LMICs, fast-tracking was needed for the establishment of SUs to revolutionize stroke care provision, with the great possibility of improving stroke outcomes [53]. Availability of designated accident and emergency departments with specific stroke clinical guidelines and functional and standardized multidisciplinary teams for acute stroke care were associated with good stroke care practice [45]. But the LMICs are grappling with the number and variety of stroke specialists, for example, very few neurologists and almost no speech therapists to form and run efficient teams [45]. There is a need for those countries to develop a competitive edge through provision of equal opportunities for career advancement, lifelong learning, and to develop policies that promote loyalty and retention [54]. Further on, this study highlighted that most LMICs countries, had inadequate facilities for neuroimaging and thrombolysis, inadequate ambulance services especially in rural areas, limited education efforts and awareness about stroke, inadequate training of emergency physicians [40], and inadequate public insurance or financial support for rehabilitation care [34]. There is a pressing need for health care providers to lobby and secure direct health policy support from the state or national level for stroke care [45] to minimize these challenges.

Six dimensions of stroke care processes that emerged in this review were assessment and diagnosis, referral, intravenous thrombolysis, rehabilitation, and primary and secondary prevention strategies [33–35, 41, 42, 46–52]. Undoubtedly, the structural limitations in LMICs has a bearing on stroke care stroke processes. This review showed that stroke assessment and diagnosis is challenged by inadequate availability of neuroimaging [40, 41]. The role for intravenous thrombolysis as evidence-based treatment for acute ischemic stroke is known [34]. It is cost-effectiveness even in LMICs [49] via its generation of optimal patient outcomes in Africa [50], and that, lack of the same adds to the existing shortage of resources [47]. We, therefore, share the observation that there is a great need for ministries of health and governments to embrace “the trends”, strengthen radiology departments and adopt thrombolysis therapy in stroke care protocols. What was also agreeable in this review was the common finding that rehabilitation represents a key part of stroke care, although, clearly, the interventions remain extremely limited in most LMICs. Research recommends that rehabilitation should commence as early as possible after stroke, within 24 to 48 h [46]. However, access to rehabilitation services remains low in LMICs, due, mostly to structural roadblocks such as limitation in number and variety of rehabilitation professionals [40, 41]. This review highlighted the need for a two-tier stroke-prevention strategy: primary preventive approaches for stroke, which included blood pressure control and promotion and maintenance of healthy lifestyle [33], and secondary prevention strategies such as blood pressure control, antiplatelet and lipid-lowering therapy, homocysteine-lowering therapy, self-management, and family support [34]. Primary preventive strategies remained key to the prevention of stroke and must be embraced in all efforts aimed at improving stroke care.

The last six dimensions that emerged were stroke care outcomes that included quality of stroke care practice, functional independence, LOS, mortality, living at home, and institutionalization. Of note was that the LMICs with better structure and processes of care such as the availability of a SU, patients were more likely to be alive, independent, and living at home 1 year after stroke [34]. Comparatively, countries with no requisites had consequent poor stroke service delivery [47]. In the LMICs where thrombolysis was administered in combination with mechanical thrombectomy there was significant improvement in functional outcome [34, 45]. Further, rehabilitative therapy was associated with reduced LOS [52], and reduced mortality [38]. On the other hand, reduced level of function was reported in the situations of absent rehabilitative treatment [39]. Additionally, where multidisciplinary teams were implemented, long-term reductions in death, dependency, and early-discharge was reported [46]. There is, therefore, a compelling need for improved structure: allocation of resources, appropriately trained personnel, and recognition of the importance of an integrated team approach to the delivery of stroke care in LMICs. This will ultimately lead to the improved delivery of stroke care [41]. and significantly improved outcomes. Many lives could be saved and the quality of life improved for the current and future generations.

This scoping review focused on a broad research field of stroke care. And 2010 to 2017 is reasonably a wide range of publication year, having only 20 articles as meeting inclusion criteria sounds spurious. However, the included articles were very specific to the study question and so could be reflective of stroke care in LMICs. Lack of strict adherence to methodological quality that goes with scoping review might affect the quality of this review. Constant checks with research team members added rigor to the study process. Additionally, the study precluded addressing issues related to personnel availability, program funding, and training institutions across the LMICs to train stroke care specialists.

## Conclusions

Inconsistencies exist in the way stroke care is advanced in LMICs. This is reflected in unsatisfactory stroke care structure, processes and outcomes in some settings of study area. There is a need for stroke care settings to adopt stroke care quality improvement strategies. Health ministry and governments need to decisively face stroke burden by setting policies that advance improved care of patients with stroke [45]. SUs and rtPA administration could be considered as both a structural and process necessity. It is clearly an imperative in LMICs such as Malawi for the care of patients with acute stroke, whose numbers are increasing exponentially.

## Data Availability

All data generated or analyzed during this study are included in this manuscript. Specific articles that were analyzed, if needed, can be obtained from the corresponding author.

## Abbreviations

CINNAHL: Cumulative Index to Nursing and Allied Health Literature
CT: Computed Tomography
DM: Donabedian Model
LMICs: Low- and Middle-Income Countries
LOS: Length of Hospital Stay
MESH: Medical subject headings
MRI: Magnetic Resonance Imaging
mRs: Modified Rankin scale
PT: Physiotherapy
rtPA: Recombinant Tissue Plasminogen Activator
SPO: Structures, Processes, and Outcomes
SU: Stroke Units
